# Deep learning for aspect-based sentiment analysis: a review

**DOI:** 10.7717/peerj-cs.1044

**Published:** 2022-07-19

**Authors:** Linan Zhu, Minhao Xu, Yinwei Bao, Yifei Xu, Xiangjie Kong

**Affiliations:** College of Computer Science and Technology, Zhejiang University of Technology, Hangzhou, China

**Keywords:** Deep learning, Aspect-based sentiment analysis, Relation extraction, Multi-task learning

## Abstract

User-generated content on various Internet platforms is growing explosively, and contains valuable information that helps decision-making. However, extracting this information accurately is still a challenge since there are massive amounts of data. Thereinto, sentiment analysis solves this problem by identifying people’s sentiments towards the opinion target. This article aims to provide an overview of deep learning for aspect-based sentiment analysis. Firstly, we give a brief introduction to the aspect-based sentiment analysis (ABSA) task. Then, we present the overall framework of the ABSA task from two different perspectives: significant subtasks and the task modeling process. Finally, challenges are proposed and summarized in the field of sentiment analysis, especially in the domain of aspect-based sentiment analysis. In addition, ABSA task also takes the relations between various objects into consideration, which is rarely discussed in the previous work.

## Introduction

With the rapid development of the Internet, user-generated content (*e.g.*, reviews) on various platforms is increasing with geometric series, which contains valuable information that helps public opinion monitoring, information prediction, user comment analysis and decision-making (Zhao, Qin & Liu, 2010; Zhu et al., 2022). Sentiment analysis (SA) is an effective method to extract this valuable information from massive data. For example, sentiment analysis of product reviews under e-commerce platforms can influence users’ desire to purchase and help enterprises improve the quality of products; sentiment analysis of event comments under social platforms can help relevant organizations discover current and future hot spots, take measures to guide.

Based on the granularity of the processing text, sentiment analysis can be divided into three different levels: document, sentence and aspect. Document- and sentence-level sentiment analysis, known as coarse-grained sentiment analysis, judge the overall sentiment polarity of the given opinion target. However, when the opinion target contains several aspects with conflict sentiment, using a unified sentiment label to represent it may be inappropriate. For example, if a laptop consists of a good screen and a poor keyboard, it can be hard to identify whether the laptop is good or not. Therefore, aspect-based sentiment analysis (ABSA), also named aspect-level sentiment analysis or fine-grained sentiment analysis, is proposed to solve the problem, which is also the purpose of this article.

In recent years, deep learning (DL) has made breakthroughs and applications in academics and industries, attracting scholars to apply it to ABSA tasks. Instead of machine learning approaches that rely heavily on the quality of the handmade features, deep learning approaches utilize neural networks (NN) to learn the semantic and syntactic features automatically and perform great in the related extensive experiment. Existing literature (Zhang, Wang & Liu, 2018; Do et al., 2019; Liu et al., 2020) has already studied the application of deep learning for ABSA, it provided a comprehensive survey of deep learning’s current application in sentiment analysis, which is organized and compared from two different perspectives: (1) subtasks of ABSA; (2) typical types of neural network architectures, mainly convolutional neural networks (CNN), recurrent neural network (RNN) and memory network (MemNN).

This article organizes the existing research work and provides a comprehensive introduction to ABSA based on deep learning. This article can help relevant researchers understand the current state of the field, and help newcomers who are interested in the field of ABSA to quickly understand. Specifically, this article describes the ABSA task from different perspectives: (1) subtasks of ABSA. Distinct from previous surveys, this article redivides the ABSA task into information extraction (IE) and sentiment classification (SC). (2) process of task modeling, where ABSA task can be divided into the form of pipeline and multi-task. Based on the perspectives above, this article further compares and analyzes the existing literature. In addition, we also discuss how relations affect the performance of the model and the challenge faced by the ABSA task.

The remainder of this article is organized as follows: the “Survey Methodology” introduces the searching process method for the literature review; “Related Concepts” introduces some preparatory knowledge for the ABSA task; “Aspect-Based Sentiment Analysis (ABSA)” presents the overall framework for the ABSA task; “Information extraction, Sentiment classification and Multi-task ABSA” discuss the specific contents of information extraction task, sentiment classification task and multi-task ABSA, respectively; “Challenges” discusses challenges for ABSA and SA; “Summary” draws the overall conclusion.

## Survey Methodology

Aiming at the theme of “deep learning for aspect-based sentiment analysis”, we searched and organized previous research articles, so as to better understand its evolution.

Firstly, we clarified research targets. Therefore, the searching process was focused on solving the following problems and challenges:

- What is aspect-based sentiment analysis?
- Why is it important to focus on aspect-based sentiment analysis?
- Where will new researchers concentrate on creating a new approach?
- How can the aspect-based sentiment analysis achieve more accurate and robust algorithms under the complex environment?

where the first two questions will prove the importance of aspect-based sentiment analysis, and the last two questions aim to help the researcher focus on the direction of the proposed approaches and achieve better algorithms.

Then, we searched the scientific articles which included the following keywords: “sentiment analysis” OR “aspect-level sentiment analysis” OR “aspect-based sentiment analysis” AND “aspect term extraction” AND “opinion term extraction” AND “sentiment classification” AND “end-to-end aspect-based sentiment analysis”. Only peer-reviewed articles written in English and published in well-known conference and online databases were included in the analysis. Then we filtered out those articles that did not reflect the deep learning approach.

**Figure 1:**
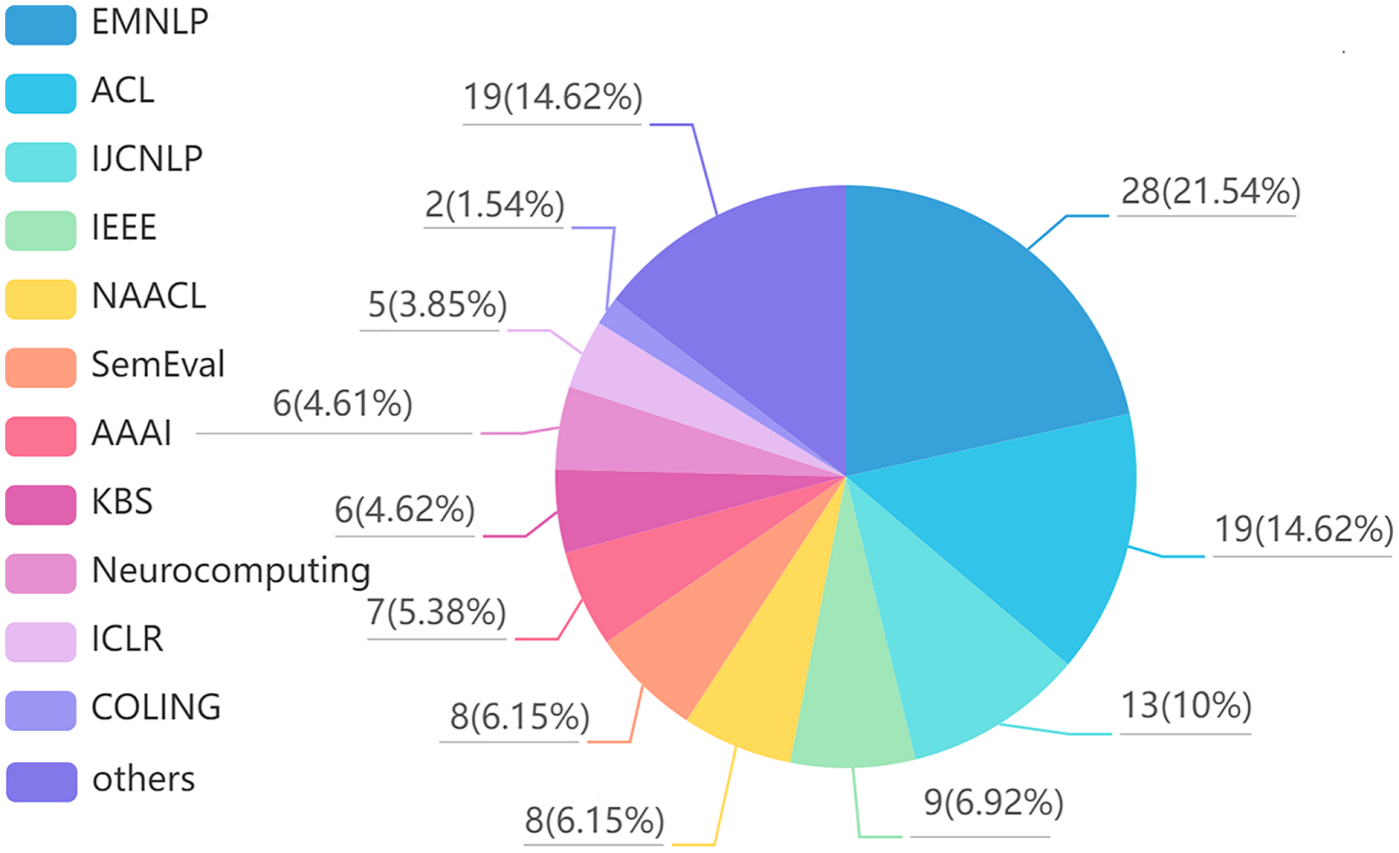
Reviewed article distribution over defined period.

**Figure 2:**
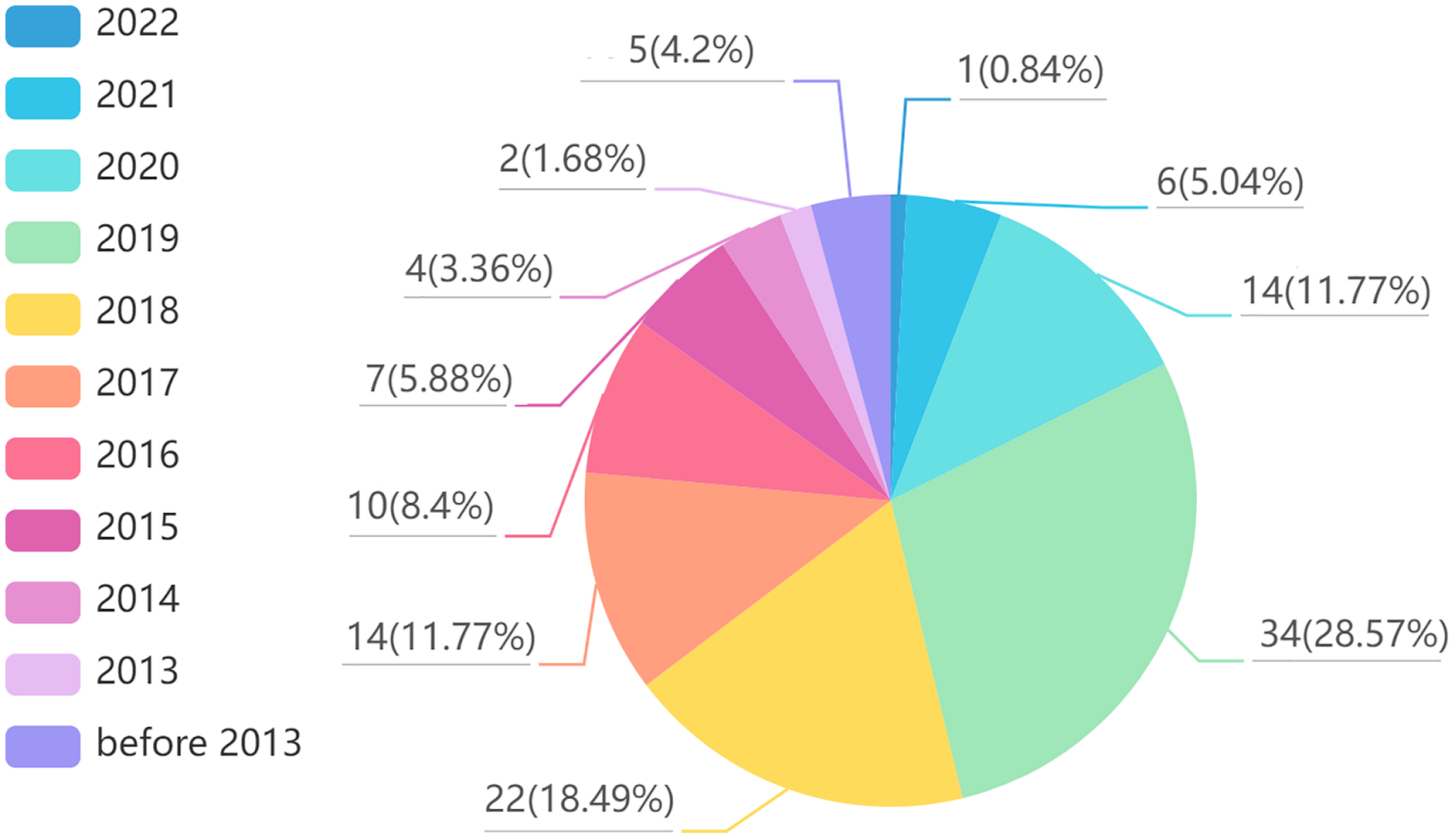
Reviewed article distribution over selected search conference and databases.

Finally, we summarized the collected articles, and the results are shown in Figs. 1 and 2. Figure 1 shows the distribution of reviewed articles over defined period. It can be seen that the review articles are mainly concentrated in 2018–2021, where aspect-based sentiment analysis was still in the development stage. Figure 2 shows the distribution of reviewed papers over selected search conference and databases. We can observe that the review articles are mainly collected from top conferences and journals (*e.g.*, ACL, NAACL, IJCNLP, IEEE, EMNLP), which ensures the authority of the review.

## Related concepts

### Deep learning (DL)

Deep learning can learn the intrinsic connections and potential patterns among large amounts of data, where deep neural networks (DNN) is the fundamental neural network architecture and many variants of DNN arise.

#### Deep neural networks (DNN)

**Figure 3:**
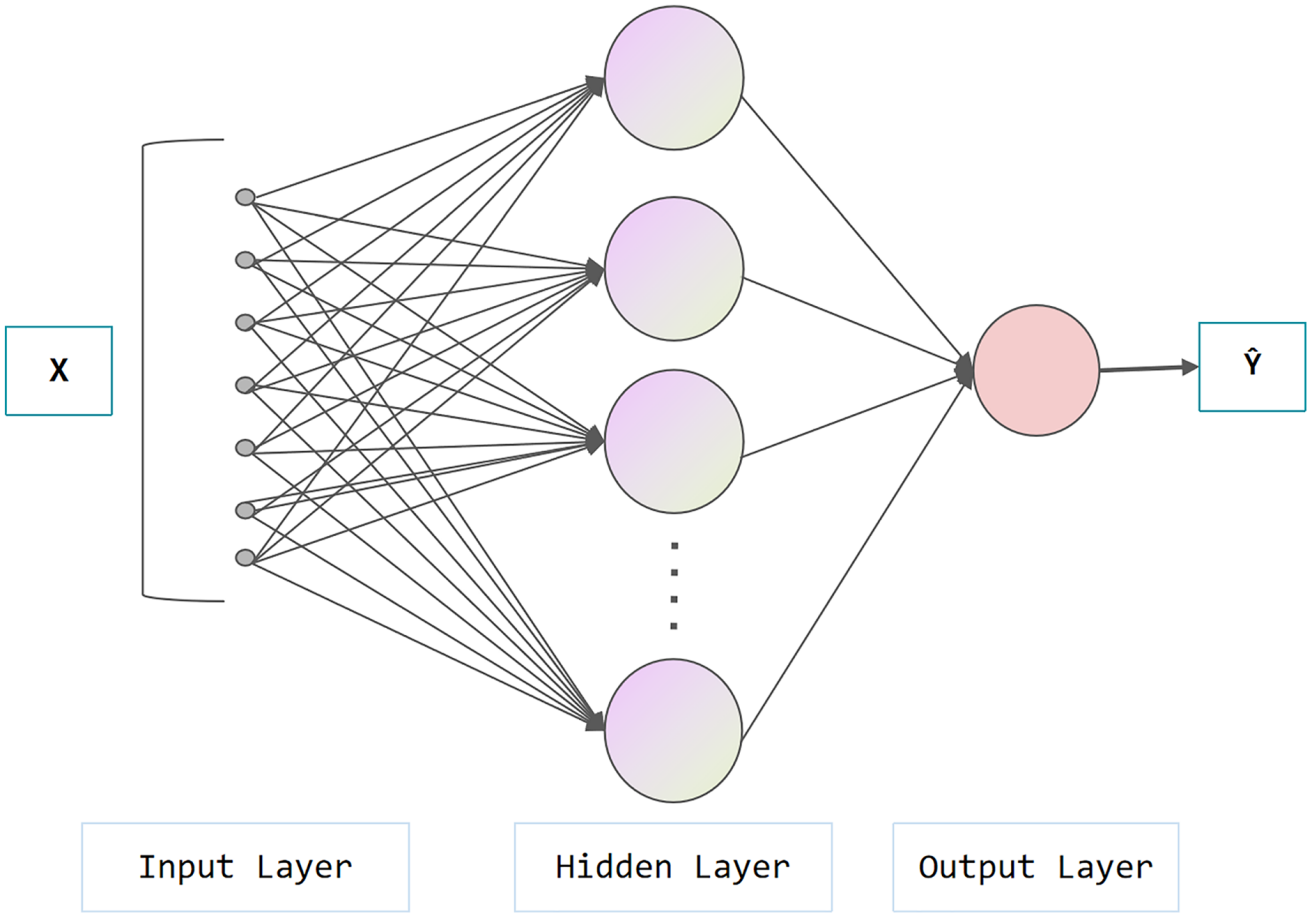
Architecture of neural network.

**Figure 4:**
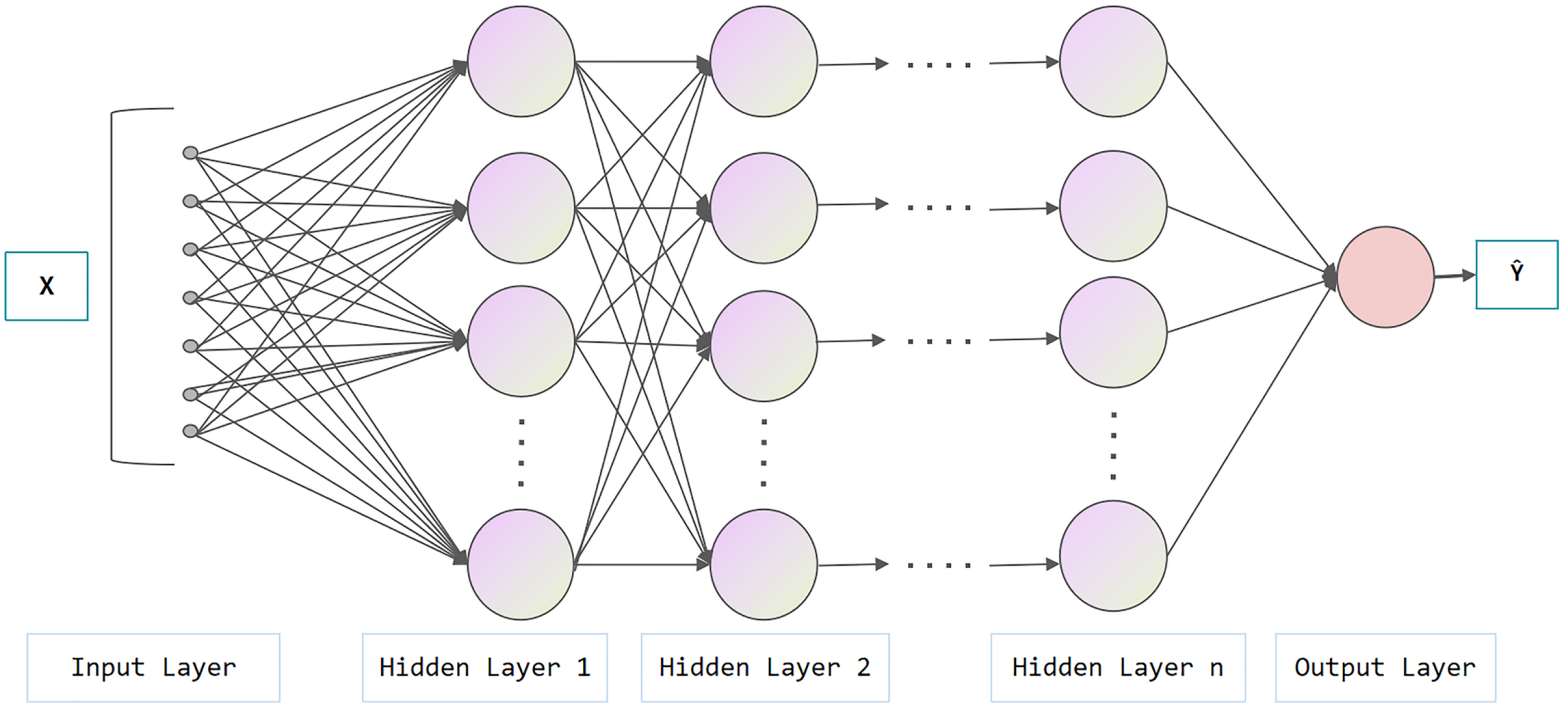
Architecture of deep neural network.

Figure 3 represents the architecture of the neural network, which is composed of an input layer, a hidden layer and an output layer. In the hidden layer, data from the input layer is added linearly and transformed nonlinearly with activation functions (*e.g.*, Sigmoid, ReLu, tanh). The hidden layer output performs similar operations at the output layer. The aforementioned process is called forward propagation, while the opposite process is called backward propagation. In the backward propagation process, the model adopts the gradient descent method and updates the parameters by minimizing the loss function, which can be used for subsequent prediction. As shown in Fig. 4, the difference between NN and DNN is that DNN has more hidden layers (at least two layers theoretically). The main idea of DNN is to regard the output of the previously hidden layer as the input of the current hidden layer to obtain more abstract high-level features.

In order to avoid gradient disappearance or gradient explosion, weight initialization in neural network is also an important task. Existing initialization approaches mainly include uniform distribution initialization, Gaussian distribution initialization, Xavier initialization (Glorot & Bengio, 2010), Kaiming initialization (He et al., 2015). Liu, Liu & Zhang (2022) proposed that an improved and extended weight initialization method with asymmetric activation function, which can expand the selection range of the activation function and improve the performance of the network.

#### Convolutional neural networks

Convolutional neural networks (CNN) (Lecun et al., 1998; Krizhevsky, Sutskever & Hinton, 2017) have the property of position invariance, local perception, weight sharing and down-sampling, which helps capture local key features. It mainly consists of an input layer, a convolution layer, a pooling layer and a full connection layer. A convolution layer and a pooling layer can be regarded as a component for feature extraction, the superposition and combination of multiple components can gradually extract higher-level features. In addition, CNN can reduce the noise introduced by the attention mechanism. For example, the attention mechanism focuses on the words “well” and “good” in the sentence “well, it’s a good idea.”, it will introduce noise as the word “well” is a mood particle and doesn’t express any sentiment.

#### Recurrent neural network

The recurrent neural network (RNN) is a sequence model which can capture long-distance dependencies. The main idea of RNN is that the current hidden state is represented by the previous hidden state and current input. However, RNN processes text from front to back, which may ignore important information followed, so BiRNN (Schuster & Paliwal, 1997) was proposed to processes text from both sides. Since the gate mechanism controls important information flow to the gate and captures long-distance dependencies which are not removed over time, long short-term memory (LSTM) (Hochreiter & Schmidhuber, 1997) and gated recurrent unit (GRU) (Cho et al., 2014) were proposed to solve gradient problem caused by the multiplicative effect in gradient backpropagation. In practice, the gradient explosion is usually solved by gradient clipping.

#### Memory neural network

Memory neural network (MemNN), mainly divided into strongly supervised (Weston, Chopra & Bordes, 2015) and weakly supervised (Sukhbaatar et al., 2015), uses external storage for a certain degree of self-supervised training to learn better embedding representations. Therefore, it has strong context awareness and processing capabilities and higher requirements for hardware.

#### Graph neural network

Graph neural network (GNN) and its variants (*e.g.*, graph convolutional networks (GCN) (Kipf & Welling, 2017), graph attention network (GAT) (Velickovic et al., 2018)) apply graph structure to the neural network. Its basic idea is to continuously use the hidden state of neighbor nodes at the current moment as part of the input to generate the hidden state of the central node at the next moment, the process is repeated until the hidden state of each node is less than a threshold. Since text can be transformed into a graph structure, GNN can fully utilize its sentence syntactic structure and word dependency. However, the information of each node in the graph is unbalanced. Xia et al. (2022) proposed a novel centrality-based framework, which uses a hub attention mechanism to assign new weights to connected non-hub vertices based on their common information with hub vertices.

### Aspect-based sentiment analysis (ABSA)

#### Definition

Sentiment analysis (SA) aims to identify people’s sentiment (*e.g.*, positive, negative, neutral) towards opinion targets (*e.g.*, products, events, comments), which expresses through opinions. Liu (2015) formulated opinions as a quadruple (*g*, *s*, *h*, *t*), where *g* represents the opinion target, *s* represents the sentiment contained in *g* and *h* represents the opinion holder, *t* represents the time when the opinion was published.

Considering the definitions above, ABSA aims to find all opinion quadruples in the given document, where the opinion target *g* in the quadruple focuses more on the specific aspect of the target instead of merely the target itself. However, most existing work focuses on the two-tuple (*g*, *s*), only a few work focuses on *h* and *t*. For example, the sentence “The food is so good and so popular that waiting can really be a nightmare.” contains aspects (“food” and “waiting”) and their corresponding sentiment (positive and negative).

#### Dataset

With the development of aspect-based sentiment analysis, the *corpus* is increasingly crucial for model construction and training. The SemEval series dataset released by the International Workshop on Semantic Evaluation is a common baseline dataset, which contains the training data for the ABSA task (Pontiki et al., 2014; Pontiki et al., 2015; Pontiki et al., 2016). Table 1 shows the detail of SemEval dataset used for ABSA task, which covers different domains (*e.g.*, restaurants, laptops) and languages (*e.g.*, English, Chinese). It is worth noting that a review is a person’s view of the opinion target and can contain several sentences.

**Table 1:** Details of SemEval dataset for ABSA.

No	Dataset	Domain	Language	Train		Test	
				Reviews	Sentences	Reviews	Sentences
1	SemEval14 Task 4	Restaurants	English	–	3,041	–	800
		Laptops	English	–	3,045	–	800
2	SemEval15 Task 12	Restaurants	English	254	1,315	96	685
		Laptops	English	277	1,739	173	761
		Hotels	English	–	–	30	266
3	SemEval16 Task 5	Restaurants	English	350	2,000	90	676
			Dutch	300	1,711	100	575
			French	335	1,733	120	696
			Russian	302	3,490	103	1,209
			Spanish	627	2,070	286	881
			Turkish	300	1,104	39	144
		Laptops	English	450	2,500	80	808
		Hotels	Arabic	1,839	4,802	452	1,227
		Mobile Phones	Chinese	140	6,330	60	3,191
			Dutch	200	1,389	70	308
		Digital Cameras	Chinese	140	5,784	60	2,256

Since most sentences in the existing ABSA dataset contain multiple aspects with the same sentiment polarity or only one aspect, the ABSA task degenerates into sentence-level sentiment analysis, Jiang et al. (2019) proposed a large-scale multi-aspect multi-sentiment dataset MAMS, which contains data for both ABSA and ACSA task. Table 2 shows the detail of MAMS, where the smaller training set can compare with the SemEval dataset.

**Table 2:** Details of the MAMS dataset.

	Dataset	Positive	Negative	Neutral	Total
ATSA	Train	3,380	2,764	5,042	11,186
	Train (small)	1,089	892	1,627	3,608
	Validation	403	325	604	1,332
	Test	400	329	607	1,336
ACSA	Train	1,929	2,084	3,077	7,090
	Train (small)	1,000	1,100	1,613	3,713
	Validation	241	259	388	888
	Test	245	263	393	901

#### Evaluate metric

The evaluate metric of ABSA is precision (P), recall (R), F1-score (F1) and accuracy (Acc).

(1) $$P = \displaystyle{{TP} \over {TP + FP}}$$

(2) $$R = \displaystyle{{TP} \over {TP + FN}}$$

(3) $$F1 = \displaystyle{{2PR} \over {P + R}}$$

(4) $$Acc = \displaystyle{{TP + TN} \over {TP + FN + FP + TN}}$$

Specifically, true positive (TP) means prediction is positive and label is positive, false negative (FN) means prediction is negative and label is positive, false positive (FP) means prediction is negative and label is positive, true negative (TN) means prediction is negative and label is negative.

### Attention mechanism

Attention mechanism means that people will pay different attention to different aspects of the opinion target, which is widely used in many fields (*e.g.*, computer vision (CV), natural language processing (NLP)).

The main idea of the attention mechanism is to find out the crucial information of the task. Specifically, given a query *Q* and a series of key-value pairs {(*K*_1_, *V*_1_), …, (*K*_*n*_, *V*_*n*_)}, attention mechanism aims to calculate the similarity between the query and each key, then sum up with a corresponding value to obtain the corresponding attention weight. The relevant formula is as follows:

(5) $$Attention(Q,K,V) = \sum\limits_{i = 1}^L Similarity(Q,{K_i})*{V_i}$$

### Word embedding

Word embedding is a vector of real numbers in a predefined vector space, which can represent word semantics. There are mainly two representation methods: one-hot encoding and distributed representation. One-hot encoding represents a word as a vector, where the dimension is the size of the vocabulary, the word’s corresponding position is set to 1, and others are all set to 0. Due to the definition of one-hot encoding, vocabulary words are independent of each other, so one-hot encoding can’t express the semantic relevance between words. Besides, the vector’s dimension depends on the vocabulary size, large vocabulary may cause sparse matrix and dimension disaster. Therefore, distributed representation (*e.g.*, Word2Vec (Mikolov et al., 2013), GloVe (Pennington, Socher & Manning, 2014), ELMo (Peters et al., 2018), BERT (Devlin et al., 2019)) is proposed to address these problems, which is low-dimensionality and can express the semantic correlation between words.

In addition, some improvements toward word embedding help ABSA tasks to obtain better performance. Akhtar et al. (2018) minimized the influence of data sparsity by using bilingual word embedding (obtained by learning a bilingual parallel *corpus*), that is, replacing the out of vocabulary (OOV) word with the nearest translation form in the shared vector space. Liang et al. (2019a) used the sparse coefficient vector to select highly relevant words, based on which the embedding representation of the target and aspect can be adjusted.

## Aspect-based sentiment analysis (absa)

**Figure 5:**
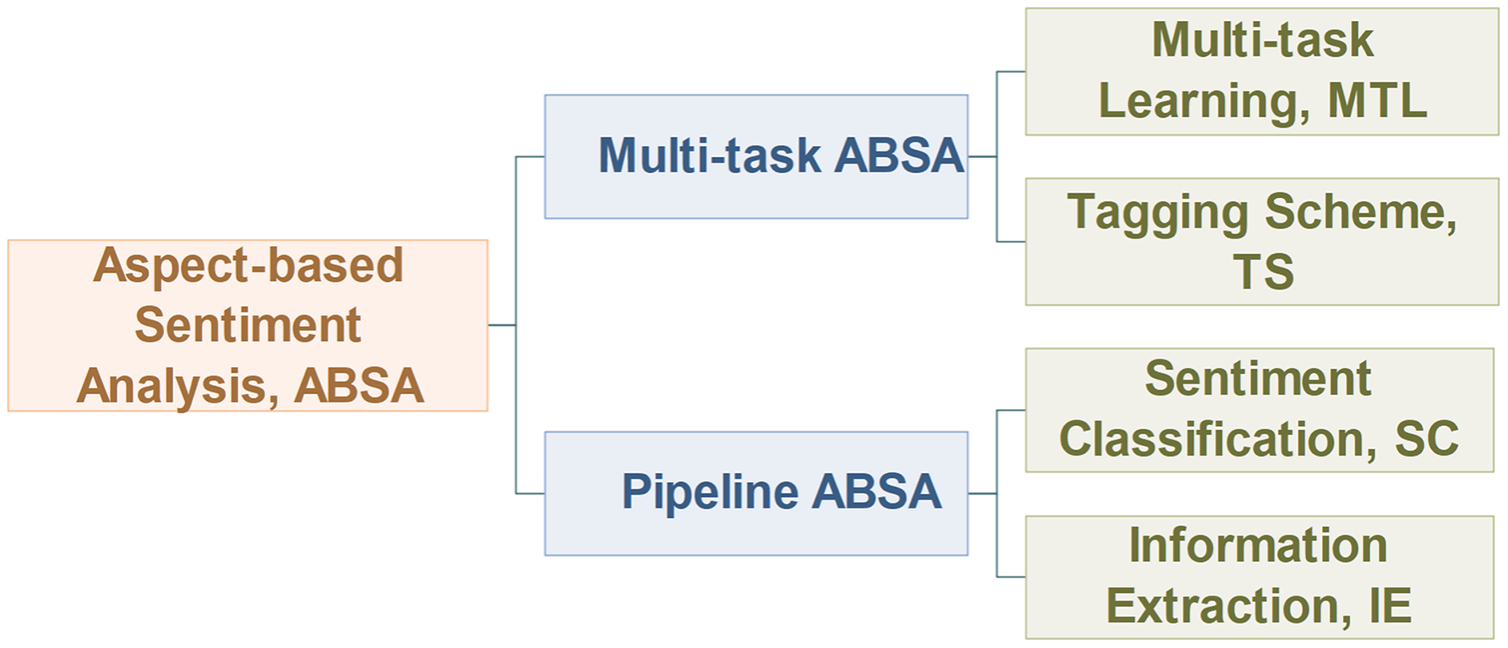
Overview of aspect-based sentiment analysis.

This article will discuss the ABSA task from two different perspectives: important subtasks and the task modeling process, as outlined in Fig. 5.

**Figure 6:**
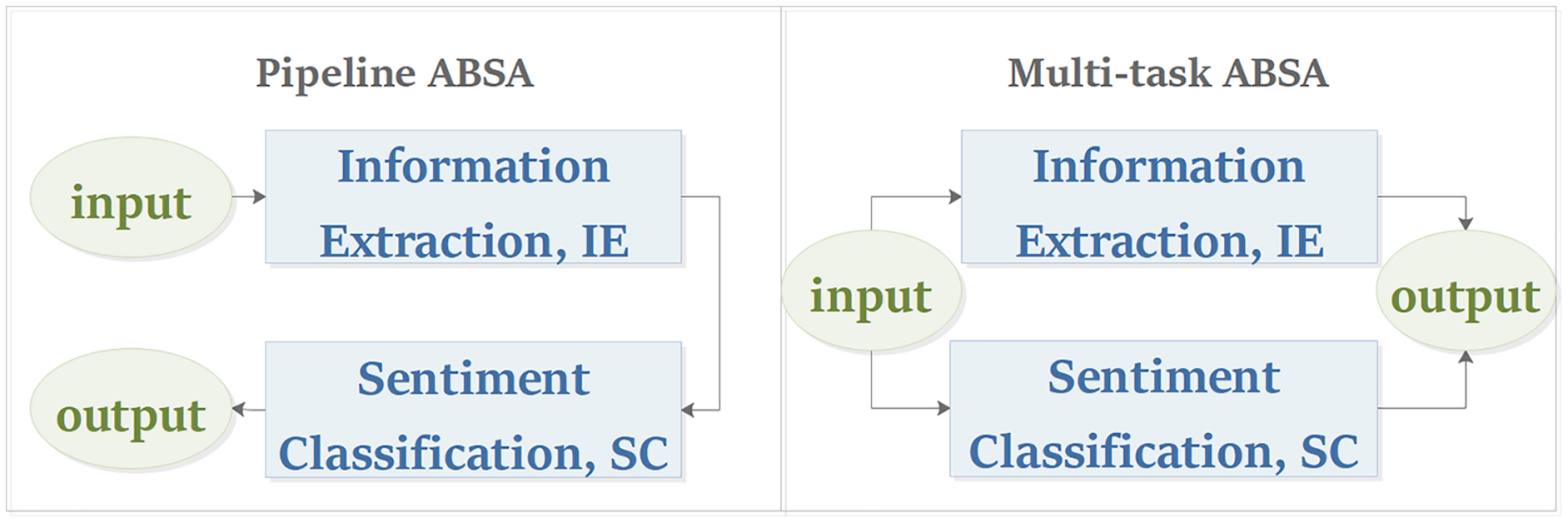
Task modeling process of aspect-based sentiment analysis.

Similar to the work of Mitchell et al. (2013), this article classifies the ABSA task into pipeline manner and multi-task manner from the perspective of the task modeling process. As shown in Fig. 6, the pipeline model processes the subtasks of ABSA in sequence, while the multi-task model processes its subtasks in parallel. For the pipeline ABSA, the main challenge is how to divide the ABSA task into reasonable subtasks. For the multi-task ABSA, the main challenges are how to learn the interactive relations between tasks from multi-task learning and how to represent the results of different types of tasks.

To solve the problem above, we re-division the ABSA task into the IE task and the SC task for the pipeline ABSA, and we also introduce some novel tasks and tagging schemes for the multi-task ABSA, which will be discussed in “Information extraction, Sentiment classification and Multi-task ABSA”.

## Information extraction (ie)

**Figure 7:**
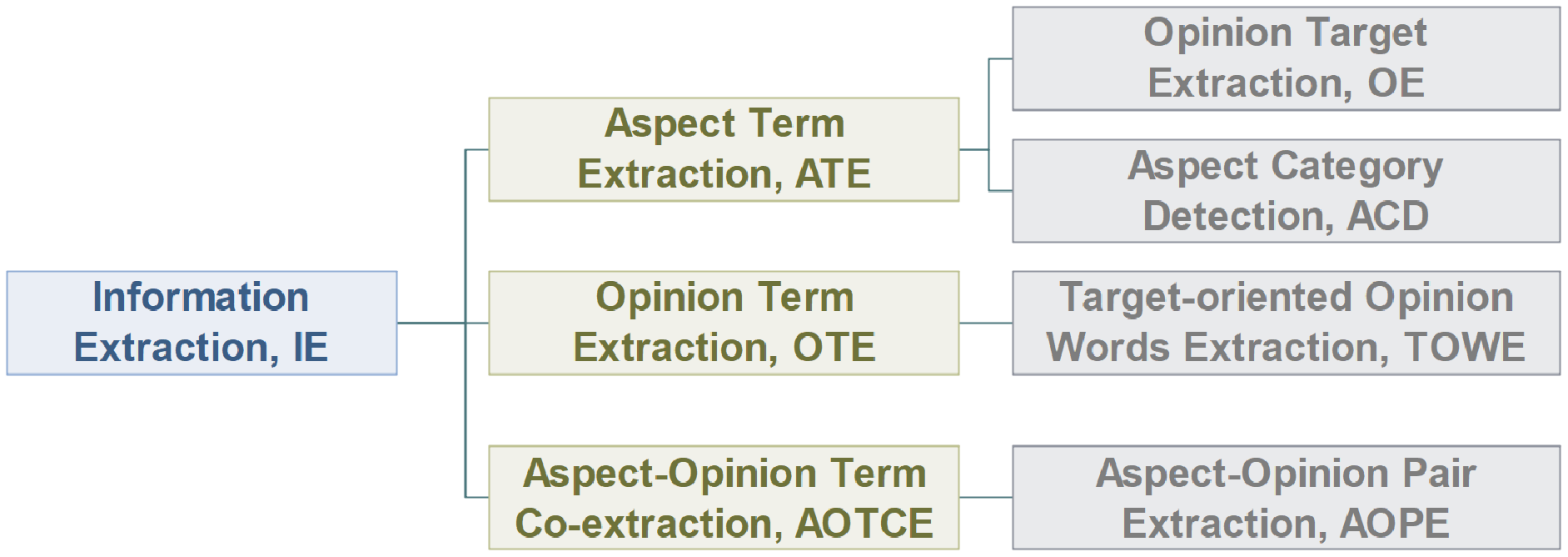
Classification of information extraction task.

Information Extraction (IE) aims to extract crucial information from the *corpus*. Current works regard it as a sequence labeling problem, which uses BIO tagging scheme {B-begin, I-inside, O-outside} or BIOES tagging scheme {B-begin, I-inside, O-outside, E-end, S-single} to perform classification on each token. As shown in Fig. 7, IE can be classified into aspect term extraction (ATE), opinion term extraction (OTE), and aspect-opinion term co-extraction (AOTCE).

### Aspect term extraction (ATE)

Aspect term extraction (ATE) can be defined as follows: Given a sentence with *n* words *S* = (*x*_1_, *x*_2_, …, *x*_*n*_) and we denote *L*_*a*_ = {B-AT, I-AT, O-AT} as the output label. For each word *x*_*i*_, the task aims to predict its corresponding label *a*_*i*_ ∈ *L*_*a*_. Most previous researches were implemented based on topic models (Shams & Baraani-Dastjerdi, 2017) and conditional random field (CRF) (Jakob & Gurevych, 2010; Shu, Xu & Liu, 2017). However, these methods have their limitations: CRF requires lots of features to work well and topic models usually do not produce highly consistent aspect terms. Therefore, deep learning methods have been proposed to address these shortcomings.

ATE task can be classified into two subtasks: opinion target extraction (OE) and aspect category detection (ACD). For example, for the sentence “The food is so good and so popular that waiting can really be a nightmare.”, the OE task extracts aspect terms (“food” and “waiting”), and the ACD task extracts aspect category (“food” and “service”). Many studies have researched the ATE task (Table 3).

**Table 3:** Application of deep learning in ATE task.

No	Study	DatasetDomain	Model	Performance			
				OE-acc	OE-F1 (%)	ACD-acc (%)	ACD-F1 (%)
1	Poria, Cambria & Gelbukh (2016)	SemEval Res14	CNN	–	86.20	–	–
			CNN+LP^1^	–	87.17	–	–
		SemEval Lap14	CNN	–	81.06	–	–
			CNN+LP	–	82.32	–	–
2	Tran, Hoang & Huynh (2019)	SemEval Res14	BiGRU-CRF	–	85	–	–
		SemEval Lap14		–	78.5	–	–
3	Xu et al. (2018)	SemEval Lap14	DE-CNN	–	81.59	–	–
		SemEval Res16		–	74.37	–	–
4	Wu et al. (2018)	SemEval Res14	unsupervised model	–	76.15	–	–
		SemEval Lap14		–	60.75	–	–
5	Ma et al. (2019)	SemEval Lap14	Seq2Seq4ATE	–	80.31	–	–
		SemEval Res16		–	75.14	–	–
6	Liao et al. (2019b)	SemEval Res14-16	LCC+GBC^2^	26.0%	41.2	–	–
		SemEval Lap14, 16		33.7%	36.1	–	–
7	Xue et al. (2017)	SemEval Res14	MTNA	–	83.65	–	88.91
		SemEval Res15		–	67.73	–	65.97
		SemEval Res16		–	72.95	–	76.42
8	Wu et al. (2019)	Digital QA reviews	MTA	–	65.67	74.92	79.65
		Beauty QA reviews		–	58.06	56.46	69.92
		Luggage QA reviews		–	63.74	63.58	57.74

**Notes:**

1 LP: linguistic patterns.

2 LCC+GBC: model that coupling global and local context.

Most of the existing research tends to study the OE task. Poria, Cambria & Gelbukh (2016) proposed a non-linear, supervised CNN with linguistic patterns to process OE task. Tran, Hoang & Huynh (2019) proposed a model that combined BiGRU and CRF, where BiGRU considers the long-distance dependency relationship of sentences and CRF considers the relationship of label transfer. Xu et al. (2018) employed two types of pre-training embedding (*i.e.*, general embedding and domain-specific embedding) on CNN to extract aspects. Wu et al. (2018) proposed a hybrid unsupervised method to solve OE task. Specifically, the model first uses chunk-level linguistic rules and a domain-related filter to extract candidate opinion targets, then uses the text with extracted chunks as pseudo-labeled data to train the GRU network. Ma et al. (2019) formalized ATE task as a sequence-to-sequence (Seq2Seq) learning task, gated unit network, and position-aware attention mechanism are also designed to capture semantics and dependence relationship between aspects and context. Based on the theory that the appearance patterns of aspects in the global context (sentence level) and local context (between the adjacent words) are usually different from other words, Liao et al. (2019b) coupled the global and local representations to find aspects.

Since the OE task and the ACD task are highly interrelated, some studies leverage multi-task learning methods to deal with these two tasks. Xue et al. (2017) proposed a multi-task learning model based on RNN and CNN to solve them at the same time. Wu et al. (2019) proposed a multi-task learning model based on QA-style reviews with character-level embedding and attention mechanism.

### Opinion term extraction (OTE)

OTE task extracts opinion terms from *corpus*, which are rarely studied (Table 4). Previous works are usually implemented based on a dictionary. For example, for the sentence “The food is so good and so popular that waiting can really be a nightmare.”, opinion terms (“good”, “popular”, “nightmare”) can be found by looking up the dictionary since it contains sentiment (positive, positive, negative).

**Table 4:** Application of deep learning in OTE task.

No	Study	Domain	Dataset	Model	Performance		
					P (%)	R (%)	F1 (%)
1	Fan et al. (2019)	Restaurant	SemEval14	IOG	82.85	77.38	80.02
		Laptop	SemEval14		73.24	69.63	71.35
		Restaurant	SemEval15		76.06	70.71	73.25
		Restaurant	SemEval16		85.25	78.51	81.69

Since aspect terms and opinion terms exist one-to-many correspondence, Fan et al. (2019) proposed a novel sequence labeling subtask named Target-oriented Opinion Words Extraction (TOWE). Formally, given a sentence with *n* words *S* = (*x*_1_, *x*_2_, …, *x*_*n*_) and an aspect *a*, we denote *L*_*o*_ = {B-OT, I-OT, O-OT} as output label. For each word *x*_*i*_, the task aims to predict its corresponding label *o*_*i*_ ∈ *L*_*o*_. Fan et al. (2019) proposed IOG model, which combined global context and the output of IO-LSTM to find the corresponding opinion terms, where IO-LSTM transmits aspect information and context information mutually.

### 5.3 Aspect-opinion term co-extraction (AOTCE)

In the broad sense, the AOTCE task extracts both aspect and opinion terms at the same time. With the development of demand, this task is transferred from the AOTCE task to the aspect-opinion pair extraction (AOPE) task (Table 5).

**Table 5:** Application of deep learning in AOTCE task.

No	Study	DatasetDomain	Model	Performance		
				F1-aspect (%)	F1-opinion (%)	F1-pair (%)
AOTCE task						
1	Wang et al. (2016a)	SemEval14 Restaurant	RNCRF	84.05	80.93	–
		SemEval14 Laptop		76.83	76.76	–
		SemEval15 Restaurant		67.06	66.90	–
2	Wang et al. (2017)	SemEval14 Restaurant	CLMA	82.02	84.40	–
		SemEval14 Laptop		76.58	78.34	–
		SemEval15 Restaurant		68.24	71.36	–
3	Yu, Jiang & Xia (2018)	SemEval14 Restaurant	GMTCMLA	84.50	85.20	–
		SemEval14 Laptop		78.69	79.89	–
		SemEval15 Restaurant		70.53	72.78	–
AOPE task						
1	Zhao et al. (2020)	SemEval14 Restaurant	SpanMlt	87.42	83.98	75.60
		SemEval14 Laptop		84.51	80.61	68.66
		SemEval15 Restaurant		81.76	78.91	64.68
		SemEval16 Restaurant		85.62	85.33	71.78
2	Chen et al. (2020)	SemEval14 Restaurant	SDRN	89.49	87.84	76.48
		SemEval14 Laptop		83.67	82.25	67.13
		SemEval15 Restaurant		74.05	79.65	70.94

AOTCE task processes two sequence labeling tasks for identifying both aspect terms and opinion terms separately. For example, for the sentence “The food is so good and so popular that waiting can really be a nightmare.”, the task can find aspect terms (“food” and “waiting”) and opinion terms (“good”, “popular”, “nightmare”) at the same time. Most previous works utilized the interactive information between aspect terms and opinion terms. Wang et al. (2016a) obtained high-level features with dependency-tree based on RecNN and CRF, where information double propagated between aspect and opinion terms. Wang et al. (2017) proposed a novel model, named coupled multi-layer attentions (CMLA), whose main idea is to capture the complex relations (direct and indirect) between aspect and opinion terms with tensor operations. On the basis of CLMA, Yu, Jiang & Xia (2018) designed a global inference module to capture the syntactic relations between aspect and opinion terms explicitly, which introduces multiple syntactic constraints (*i.e.*, intra-task constraints, inter-task constraints and lexicon constraints).

However, opinion terms may match the wrong aspect for the AOTCE task, which can further lead to the wrong judgment of sentiment classification. Thus, on the basis of the AOTCE task, the AOPE task is proposed to solve the problem above by pairing aspect and opinion terms. For example, we further obtain the aspect-opinion pair (“food”-“good”, “food”-“popular”, “waiting”-“nightmare”) from the example above. Zhao et al. (2020) proposed a novel model SpanMlt, which processes the sentence at span-level and contains term and relation scorer to identify aspect terms, opinion terms and its possible relations. Chen et al. (2020) designed the double-channel network to extract opinion entities and relations simultaneously, two synchronization mechanisms is proposed to make full use of extracted information.

### Summary

In this section, we discuss the subtasks of IE task—ATE, OTE and AOTCE (Fig. 7).

ATE extracts aspect terms from the *corpus*. ATE task is classified into OE and ACD according to whether the aspect terms or the aspect terms category is extracted. Existing literature usually regard ATE task as sequence labeling problem, using deep learning models to label each word as aspect words or non-aspect words. In addition, models can achieve better performance by enriching word embedding, considering highly correlation between OE and ACD tasks.

OTE extracts opinion terms from the *corpus*, which is a rarely studied task. To be simple, the TOWE task has been designed to find one-to-many correspondences between the given aspect and opinion terms.

We also discuss the evolution of the AOTCE task, which transferred from joint extraction to pair extraction. Most works about the AOTCE task propagated the interactive information between ATE and OTE tasks to obtain higher-level features, but models may mismatch aspect terms and opinion terms. So AOPE task has been designed to match aspect terms and opinion terms correctly based on the results of the AOTCE task. Table 5 shows the application of DL in the AOTCE task. We can find that the result of the AOPE task is better than AOTCE in general, its main reason is that the interaction information between ATE and OTE is considered.

## Sentiment classification (sc)

Sentiment classification (SC), which is also named sentiment polarity (SP), aims to identify the corresponding polarity of sentiment (*i.e.*, positive, negative, neutral) with information extracted from the IE task. Formally, given a sentence with *n* word *S* = (*x*_1_, *x*_2_, …, *x*_*n*_) and an aspect *a*, we denote *L*_*p*_ = {Positive, Negative, Neutral} as output label. For the aspect *a*, the SC task aims to predict its corresponding label *p* ∈ *L*_*p*_. According to the relationship between different targets (*e.g.*, aspect, context, language structure), SC can be classified into aspect-context relation sentiment classification, aspect-aspect relation sentiment classification, language structure sentiment classification and their hybrid methods. In addition, we will discuss external knowledge and tools for SC tasks.

### Aspect-context relation sentiment classification

Aspect-context relation sentiment classification mainly captures the interaction information (*e.g.*, semantic information, position information, syntax information) between aspect and context, which is used to learn aspect-specific feature representations or aspect-specific sentence representations. Table 6 shows the related work.

**Table 6:** Aspect-context relation sentiment classification.

No	Model	SemI^3^	AspR^4^	PosI^5^	SynI^6^	Dataset	Acc (%)	Macro-F1 (%)
1	ATAE-LSTM (Wang et al., 2016b)	*✓*				Res14	77.20	–
						Lap14	68.70	–
2	AF-LSTM (Tay, Tuan & Hui, 2018)	*✓*				Res14Res15	75.44	–
						Lap14Lap15	68.81	–
3	IAN (Ma et al., 2017)	*✓*				Res14	78.60	–
						Lap14	72.10	–
4	MGAN (Fan, Feng & Zhao, 2018)	*✓*	*✓*	*✓*		Res14	81.25	71.94
						Lap14	75.39	72.47
5	ReMemNN (Liu & Shen, 2020)	*✓*				Res14	79.64	68.36
						Lap14	71.58	65.41
6	PF-CNN (Huang & Carley, 2018)	*✓*				Res14	79.20	–
						Lap14	70.06	–
	PG-CNN (Huang & Carley, 2018)	*✓*				Res14	78.93	–
						Lap14	69.12	–
7	FDN (Shuang et al., 2020)	*✓*				Res14	82.30	75.00
						Lap14	76.80	72.50
8	Tensor DyMemNN (Tay, Tuan & Hui, 2017)	*✓*				Res14	–	58.61
						Lap14	–	55.24
	Holo DyMemNN (Tay, Tuan & Hui, 2017)	*✓*				Res14	–	58.82
						Lap14	–	60.11
9	Co-attention+LRBP (Zhang et al., 2019)	*✓*		*✓*		Res14	80.35	–
						Lap14	73.20	–
10	MemNet (Tang, Qin & Liu, 2016b)	*✓*		*✓*		Res14	80.95	–
						Lap14	72.21	–
11	RAM (Chen et al., 2017b)	*✓*		*✓*		Res14	80.23	70.80
						Lap14	74.49	71.35
12	TNet-LF (Li et al., 2018)	*✓*		*✓*		Res14	80.79	70.84
						Lap14	76.01	71.47
	TNet-AS (Li et al., 2018)	*✓*		*✓*		Res14	80.69	71.27
						Lap14	76.54	71.75
13	AE-DLSTMs (Shuang et al., 2019)	*✓*		*✓*		Res14	79.57	–
						Lap14	72.10	–
	AELA-DLSTMs (Shuang et al., 2019)	*✓*		*✓*		Res14	80.35	–
						Lap14	73.91	–
14	History-based soft label model (Yin et al., 2019)	*✓*		*✓*		Res14	80.98	71.52
						Lap14	74.56	71.63
15	CapsNet (Jiang et al., 2019)	*✓*		3*✓		MAMS	79.776	–
						MAMS-small	73.860	–
						Res14	80.786	–
	CapsNet-BERT (Jiang et al., 2019)	*✓*		*✓*		MAMS	83.391	–
						MAMS-small	80.910	–
						Res14	85.934	–
16	IACapsNet (Du et al., 2019)	*✓*		*✓*		Res14	81.79	73.40
						Lap14	76.80	73.29
17	SAGAT (Huang et al., 2020)	*✓*			*✓*	Res14	85.08	77.94
						Lap14	80.37	76.94
18	ASGCN-DT (Zhang, Li & Song, 2019)	*✓*		*✓*	*✓*	Res14	80.86	72.19
						Res15	79.34	60.78
						Res16	88.69	66.64
						Lap14	74.14	69.24
	ASGCN-DG (Zhang, Li & Song, 2019)	*✓*		*✓*	*✓*	Res14	80.77	72.02
						Res15	79.89	61.89
						Res16	88.99	67.48
						Lap14	75.55	71.05
19	CDT (Sun et al., 2019)	*✓*			*✓*	Lap14	77.19	72.99
						Res14	82.30	74.02
						Res16	85.58	69.93

**Notes:**

3 Semantic information.

4 Aspect-aspect relations.

5 Position information.

6 Syntax information.

Every existing work almost takes semantic information between aspect and context into consideration. Many studies use attention mechanism to integrate aspect information into context. Wang et al. (2016b) proposed attention-based LSTM with aspect embedding (ATAE-LSTM) based on attention-based LSTM (AT-LSTM), where aspect information is integrated into the input and the process of attention weight calculation. Based on ATAE-LSTM, Tay, Tuan & Hui (2018) proposed aspect fusion LSTM (AF-LSTM), which adopted cyclic convolution and cyclic correlation to model the aspect-context relationship. Ma et al. (2017) proposed interactive attention networks (IAN) to interactively learn attention weights of context and aspect to generate representations of aspect and context respectively. Based on coarse-grained attention mechanisms proposed by IAN, Fan, Feng & Zhao (2018) combined it with fine-grained attention mechanisms to better model word-level interaction between aspect and context. To tackle weak interaction in attention mechanism, Liu & Shen (2020) designed a multi-element attention mechanism to capture precise semantic relation among aspect, context, and hidden state. However, the attention mechanism pays much attention to frequent opinion terms and ignores the infrequent ones, which may introduce noise. Xue & Li (2018), Huang & Carley (2018), and Shuang et al. (2020) used filters and gate mechanism to alleviate the problem by controlling the spread of sentiment information in context. Tay, Tuan & Hui (2017) integrated parameterized neural tensor synthesis or holographic synthesis into the memory selection operation to capture binary interactions between aspect and context. Different from using aspect independent (weakly related) encoders to generate sentence representations, Liang et al. (2019b) improved feature selection and extraction capabilities under the guidance of aspects.

Some studies consider location information to achieve better performance. Target-dependent LSTM (TD-LSTM) (Tang et al., 2016a) split the text according to the position of aspect, both of which is end with aspect and process with LSTM. Target-connection LSTM (TC-LSTM) improved TD-LSTM by concatenating the aspect vector with the context vector. Similarly, Zhang et al. (2019) proposed an efficiency model, which converges at least four times faster and uses co-attention matrices to capture precise semantic relation among aspect, left context, and right context. We can observe that the closer to the aspect, the more correlation information words may contain, so location information can be obtained by assigning attention weight to different contexts according to their distance from the aspect (Tang, Qin & Liu, 2016b; Chen et al., 2017b; Li et al., 2018; Fan, Feng & Zhao, 2018; Shuang et al., 2019; Yin et al., 2019). Besides, the capsule network contains location information, which encodes the spatial relationship between features. Jiang et al. (2019) proposed CapsNet and CapsNet-BERT to encode spatial information as features. Du et al. (2019) introduced the interactive attention mechanism in the capsule routing process to model the semantic relation between aspect and context.

Some studies also consider syntactic information to achieve better performance. Current studies transformed sentences into dependency graph or dependency tree structure, then applied GNN to this structure. Instead of traditional bottom-up dependencies, Zhang & Zhang (2019) used three information exchange channels (*i.e.*, self-to-self, top-to-down, bottom-to-up) to obtain more consistent phrase-level sentiment prediction. Huang et al. (2020), Zhang, Li & Song (2019), and Sun et al. (2019) built GCN over the dependency tree to propagate sentiment features or dependency tree from important syntax neighborhood words to aspect and further exploit syntax information and long-distance word dependencies.

### Hybrid methods

In addition to aspect-context relation, existing studies improve the performance of the model by mixing other different relations (*e.g*. aspect-aspect relation, relations contained in language structure) and introducing external knowledge and tools. Table 7 shows the related work.

**Table 7:** Application of deep learning in hybrid sentiment classification methods.

No	Model	SemI^3^	AspR^4^	PosI^5^	DatasetDomain	Label classes	Accuracy (%)	Macro-F1 (%)
Task2: aspect-aspect relation sentiment classification								
	MIAD (Hazarika et al., 2018)	*✓*	*✓*		SemEval Res14	3	79.00	–
					SemEval Lap14		72.50	–
2	IARM (Majumder et al., 2018)	*✓*			SemEval Res14	3	80.00	–
					SemEval Lap14		73.80	–
Task3: language structure sentiment classification								
1	H-LSTM (Ruder, Ghaffari & Breslin, 2016)	*✓*			SemEval Res16 English	3	83.00	–
					SemEval Lap16 English		77.40	–
	ATSM-S (Peng et al., 2018a)	*✓*			Chinese datasets^7^	3	85.95	80.13
Task4: sentiment classification with external knowledge and tools								
1	Attention+WordNet (Kumar, Kawahara & Kurohashi, 2018)	*✓*			SemEval Microblog17^8^	3	79.40%	–
					SemEval News17		78.20	–
2	LSTM+SKG (Chen & Huang, 2019)	*✓*		*✓*	Chinese car review^9^	4	75.82 ± 0.82	64.33 ± 1.29
	MemNet+SKG (Chen & Huang, 2019)	*✓*		*✓*			77.45 ± 0.52	67.02 ± 0.80
	AT-LSTM+SKG (Chen & Huang, 2019)	*✓*		*✓*			77.07 ± 0.67	66.24 ± 1.53
	RAM+SKG (Chen & Huang, 2019)	*✓*		*✓*			78.74 ± 0.63	69.07 ± 0.68
3	TransCap (Chen & Qian, 2019)	*✓*		*✓*	SemEval Res14	3	79.55	71.41
					SemEval Lap14		73.87	70.10
4	CAN (Hu et al., 2019b)	*✓*			SemEval Res14	3	83.33	73.23
						2	89.02	84.76
					SemEval Res15	3	78.58	54.72
						2	81.75	80.91
	ATLX (Bao, Lambert & Badia, 2019)	*✓*			SemEval Res14	3	80.06–83.45	–

**Notes:**

3 Semantic information.

4 Aspect-aspect relations.

5 Position information.

7 Chinese aspect datasets from Che et al. (2015).

8 SemEval 2017 Task 5 Microblog from Cortis et al. (2017).

9 Chinese car review from Chen & Huang (2019).

Aspect-aspect relation usually appears in multi-aspect sentences, where aspects arranged in order will show a high degree of sentiment relevance and interaction. So it can provide sufficient sentiment classification information. Hazarika et al. (2018) generated aspect-specific sentence representations for each aspect and modeled dependency relationship between aspects according to the occurrence order. Based on Hazarika et al. (2018)’s work, Majumder et al. (2018) proposed a memory network with a multi-hop attention mechanism to obtain more accurate representation by repeatedly matching aspect-specific sentence representation with others. Besides, Fan, Feng & Zhao (2018) designed an aspect alignment loss to enhance attention weights difference among aspects.

Relations contained in language structure contain much important information, which can further help sentiment classification task. Ruder, Ghaffari & Breslin (2016) proposed a hierarchical LSTM (H-LSTM), which stacked a review-level Bi-LSTM on top of sentence-level Bi-LSTMs to model intra- and inter-sentence relations simultaneously. According to the hypothesis that aspect target sequence can control the final sentiment classification results, Peng et al. (2018a) learned Chinese aspects from three different levels of granularity: radical, character, and word.

External knowledge and tools can provide additional prior knowledge and help model focuses more on the important information. Kumar, Kawahara & Kurohashi (2018) proposed a two-layered attention network similar to H-LSTM, which generates better embedding with WordNet. Chen & Huang (2019) generated the knowledge vector with the sentimental knowledge graph. Due to the lack of aspect-level datasets and the easy access to document-level datasets, Chen & Qian (2019) proposed the transfer capsule network (TransCap), which transfers document-level knowledge to aspect-based sentiment classification tasks. Hu et al. (2019b) designed orthogonal regularization and sparse regularization and Bao, Lambert & Badia (2019) used lexicon information and attention regularizer to restrict the allocation of attention weights and provide key information. Besides, some studies (Jiang et al., 2019; Huang et al., 2020) integrated pre-trained BERT into the model to further improve the performance.

### Summary

In this section, we discuss the SC task according to the relationship between different targets (*e.g.*, aspect, context, language structure). Aspect-context relation is the most fundamental relation for sentiment classification, it learns aspect-specific feature representations or aspect-specific sentence representations with the help of the interaction information (*e.g.*, semantic information, position information, syntax information) between aspect and context. Aspect-aspect relation, language structure relations, and external knowledge and tools provide extra information, to further improve the performance of the model. Tables 6 and 7 shows the performance of proposed model above. We can find that binary classification accuracy is higher than ternary classification accuracy, because sometimes it’s difficult for neural networks to distinguish neutral sentiment. Besides, relations between different targets can help us improve the performance.

## Multi-task absa

The performance of pipeline ABSA depends on the quality of the IE task. In other words, errors produced in the IE task will affect the performance of the SC task, thus limiting the performance of the overall ABSA task (Schmitt et al., 2018). Moreover, in the pipeline ABAS, the IE task can’t use the information from the SC task, and the joint information between tasks is overlooked. Therefore, multi-task ABSA is proposed to utilize the independent and joint information of important subtasks of ABSA. The experiments have proved that it has a considerable performance improvement compared to the pipeline mode (Schmitt et al., 2018; Nguyen & Shirai, 2018). Li et al. (2019a) also investigated the effectiveness of pre-trained language models BERT, its work can serve as a BERT-based benchmark for E2E-ABSA.

With the gradual deepening of cognition, the focus of existing research has gradually shifted from pipeline manner to multi-task manner. There are mainly two challenges for the multi-task ABSA, which will be explained in detail in this section.

### Multi-task learning

The main idea of the multi-task ABSA is to capture the complex interactive relations between its important subtasks of aspect-based sentiment analysis and obtain enriched feature representations.

Multi-task ABSA can be divided into aspect sentiment pair extraction (ASPE), aspect sentiment triple extraction (ASTE), aspect sentiment quadruple extraction (ASQE). Specifically, ASPE task aims to extract opinion pair (aspect term, sentiment polarity), ASTE task aims to extract opinion pair (aspect term, opinion term, sentiment polarity), ASQE task aims to extract opinion pair (aspect term, aspect category, opinion term, sentiment polarity). In the early approaches, ATE task and SC task were considered important subtasks of multi-task ABSA, also known as the ASPE task. Luo et al. (2019) designed a cross-sharing unit (CSU) to take full advantage of the interaction between ATE and SC tasks. With the increasing demand for sentiment analysis, multi-task ABSA is no longer limited to the two important subtasks of ATE task and SC task. OTE task is also an important subtask of multi-task ABSA because aspect terms express their sentiment through opinion terms, resulting in the ASTE task. Peng et al. (2020) first proposed the ASTE task, which transformed ATE, OTE, and SC tasks into a unified task and provided a complete one-time solution. Chen & Qian (2020) proposed relation-aware collaborative learning (RACL), a multi-layer multi-task learning framework with a relation propagation mechanism, to make full use of relations between ATE, OTE, and SC tasks. Zhang et al. (2020) proposed a multi-task learning framework to jointly extract aspect terms and opinion terms and simultaneously parses sentiment dependencies and used biaffine scorer (Dozat & Manning, 2017) to capture the interaction of two words in each word pair. Chen et al. (2021b) proposed an approach by considering the rich syntactic dependence and semantic word similarity in sentences (such as self-interaction relations, neighbor relations, and dependency relations), and adopted GraphSAGE (Hamilton, Ying & Leskovec, 2017) to obtain the rich feature representation of words. Since there are too many aspect terms in the *corpus*, and the same aspect term may have different descriptions, Cai, Xia & Yu (2021) proposed ASQE task, took ACD task as an important sub-task of multi-task ABSA, and proposed four baseline methods.

### Tagging scheme

Previous studies regarded multi-task ABSA as the sequence tagging problem, which can be classified into joint mode and collapsed mode. Specifically, joint mode treats *N* important sub-tasks as *N* independent sequence tagging problems, while collapsed mode treats them as one independent sequence tagging problem. Table 8 shows the difference between joint mode and collapsed mode. Given the example sentence, assume that ATE task uses the BIO tagging scheme *BIO* = {*B* − *Begin*, *I* − *Inside*, *O* − *Outside*} and the SC task use *L* = {*POS*, *NEG*, *NEU* } to label each word, joint mode will label the word “food” “B” for ATE task and “POS” for the SC task, while collapsed mode will label “B-POS”.

**Table 8:** Difference between joint mode and collapsed mode of multi-task aspect-based sentiment analysis.

Example		The	food	is	good	.
Pipline	ATE	O	B	O	O	O
	SC	NEU	POS	NEU	NEU	NEU
multi-task		O	B-POS	O	O	O

Since the multi-task ABSA can be decomposed into many different types of sub-tasks (*e.g.*, extraction task, pair task, and classification task), how to formalize the multitask ABSA task into a unified tagging scheme is another great challenge. Some novel tagging schemes are proposed to solve the problem. In order to capture the many-to-one and one-to-many relationships between aspect terms and opinion terms, Wu et al. (2020) proposed a novel tagging scheme, grid tagging scheme (GTS), to label the relations of all word pairs with tags *C* = {*N* − *None*, *A* − *Aspect*, *O* − *Opinion*, *Pos* − *Positive*, *Neg* − *Negative*, *Neu* − *Neutral*}. Xu et al. (2020) proposed a novel tagging scheme, position-aware tagging scheme (JET), which take sentiment polarities, opinion terms and position information into consideration.

In addition, current work also translates the multi-tasking ABSA task into other forms of problems. Yan et al. (2021) transformed ASTE task into sequence to sequence (Seq2Seq) problem and solved it using BART (Lewis et al., 2020), which is a pre-trained Seq2Seq model. Mao et al. (2021) converted ASTE task into two machine reading comprehension (MRC) problems, which were solved by joint training of two Bert-MRC models with shared parameters. Chen et al. (2021a) transformed ASTE task into a multi-turn MRC (MTMRC) problem and solved it with the proposed Bidirectional machine reading comprehension (BMRC) framework. Zhang et al. (2021) regarded the ASQP task as a paraphrase generation process, using the formula “Category is Sentiment because Aspect is Opinion.” to restatement statement.

### Summary

In this section, we briefly discuss multi-task ABSA and its main challenges. Different from pipeline manner ABSA, multi-task ABSA improved the model’s generalization ability by making full use of the independent and joint information of its subtask task, which can be classified into ASPE task, ASTE task and ASQE task. Since multi-tasking ABSA encompasses multiple types of tasks, performing it within a unified framework is also a challenge. Table 9 shows the current research in multi-task ABSA.

**Table 9:** Application of deep learning for multi-task ABSA.

No	Model	Dataset	Pair-F1 (%)	Triplet-F1 (%)	Quadruple-F1 (%)
1	KHW (Peng et al., 2020)	SemEval14 Restaurant	56.10	51.89	
		SemEval14 Laptop	53.85	43.50	
		SemEval15 Restaurant	56.23	46.79	
		SemEval16 Restaurant	60.04	53.62	
2	OTE-MTL (Zhang et al., 2020)	SemEval14 Restaurant		59.67	
		SemEval15 Restaurant		48.97	
		SemEval16 Restaurant		55.83	
		SemEval14 Laptop		45.05	
3	S3E2 (Chen et al., 2021b)	SemEval14 Restaurant		66.74	
		SemEval14 Laptop		52.01	
		SemEval15 Restaurant		58.66	
		SemEval16 Restaurant		66.87	
4	Double-Propagation-ACOS (Cai, Xia & Yu, 2021)	Restaurant-ACOS			21.04
		Laptop-ACOS			8
	JET-ACOS (Cai, Xia & Yu, 2021)	Restaurant-ACOS			39.01
		Laptop-ACOS			23.81
	TAS-BERT-ACOS (Cai, Xia & Yu, 2021)	Restaurant-ACOS			33.53
		Laptop-ACOS			27.31
	Extract-Classify-ACOS (Cai, Xia & Yu, 2021)	Restaurant-ACOS			44.61
		Laptop-ACOS			35.80
5	BARTABSA (Yan et al., 2021)	SemEval14 Restaurant	77.68	72.46	
		SemEval14 Laptop	66.11	57.59	
		SemEval15 Restaurant	67.98	60.11	
		SemEval16 Restaurant	77.38	69.98	
		SemEval14 Restaurant		65.25	
		SemEval14 Laptop		58.69	
		SemEval15 Restaurant		59.26	
		SemEval16 Restaurant		67.62	
6	Dual-MRC (Mao et al., 2021)	SemEval14 Restaurant	74.93	70.32	
		SemEval14 Laptop	63.37	55.58	
		SemEval15 Restaurant	64.97	57.21	
		SemEval16 Restaurant	75.71	67.40	
7	BMRC (Chen et al., 2021a)	SemEval14 Laptop	67.45	59.27	
		SemEval14 Restaurant	76.23	70.69	
		SemEval15 Restaurant	68.60	61.05	
		SemEval16 Restaurant	76.52	68.13	
8	PARAPHRASE (Zhang et al., 2021)	SemEval15 Restaurant			46.93
		SemEval16 Restaurant			57.93

## Challenges

From the above discussion, we can see that the application of deep learning for ABSA is still in the initial stage, there are still many challenges for sentiment analysis, especially aspect-based sentiment analysis.

### Implicit sentiment analysis

There are 20% to 30% of sentimental expressions have no explicit sentimental words, so implicit sentiment analysis, which studies the sentiment of texts without sentimental words, has gradually become the focus of many scholars. Implicit sentiment is usually expressed by fact-implied and linguistic rhetoric, where 72% of implicit sentiment sentences are fact-implied ones. Specifically, fact-implied expresses the sentiment by describing the facts, while linguistic rhetoric expresses the sentiment through linguistic rhetoric, such as metaphors and rhetorical (Liao, Wang & Li, 2019a). For example, “It’s only five a.m. and music is already playing in the park.” is a fact-implied, which expresses people’s complaints about noise, because it is when people are sleeping; “He is a snake.” is a rhetorical expression, which expresses his insidious and cunning characteristics.

There have been some works on the implicit sentiment analysis task, its main challenge is how to perceive sentiment and how to integrate external knowledge into the model elegantly. Liao, Wang & Li (2019a) analyzed fact-implied implicit sentiment and found it is usually affected by sentiment target, context semantic background and sentence structure, then a multi-level semantic fusion method is proposed to learn the above features. Wei et al. (2020) proposed a BiLSTM model with multi-polarity orthogonal attention to capture the differences between words and sentiment polarity, orthogonal restriction is also designed to maintain the difference during optimization. Su, Tian & Chen (2016) describes an algorithm to interpret the Chinese nominal and verbal metaphors based on latent semantic similarity, where latent semantic similarity is based on WordNet. Peng, Su & Chen, 2018b) proposed target attention-based LSTM (TRAT-LSTM), which introduced the interaction between aspect and context to obtain reliable sentiment-related classification features. Based on TRAT-LSTM, Su et al. (2019) constructed a cultural-related aspect lexicon and proposed culture-related attention-based LSTM (CRAB-LSTM), which also considered the knowledge of Chinese culture. Mao, Lin & Guerin (2019) using linguistic theories (MIP and SPV) to directly inform the design of DNN for end-to-end sequential metaphor identification. Bouazizi & Ohtsuki (2016) proposed a pattern-based approach and four sets of features to detect sarcasm on Twitter.

### Domain adaptation

Sentiment classification is widely known as a domain-dependent problem. In order to learn and obtain an accurate domain-specific sentiment classifier, a large number of labeled samples are needed, which are expensive and time-consuming to annotate (Yuan et al., 2018). Moreover, different datasets in different fields are often in great differences, which makes the effects applied to different fields uneven. Domain adaption methods transform learned knowledge or patterns from a certain domain to a new domain, which requires less supervised training data.

Mining commonalities and differences between domains is a typical method to complete the task. With the help of domain knowledge, Yuan et al. (2018) modeled the task at the feature-level and selected the most domain-related sentimentally discerning features in the sharing the feature extraction layer. Although domain-specific information is not transferable, learning domain-specific representation can help to learn domain-invariant representations, Hu et al. (2019a) used an orthogonal domain-related task (*e.g.*, ATE task) to obtain domain-specific information, so as to further extract domain-invariant features.

Same expressions towards different aspects in different domains may produce different sentiments, so it’s necessary to bridge the gap between domains and find more discriminative features. Wang & Pan (2018) effectively reduced word-level domain offsets with syntactic relations, which are also used as cross-domain invariant “axis information” to establish structural correspondences, then an auxiliary task is generated to predict the syntactic relations between any two adjacent words in the dependence tree. Hu et al. (2019a) designed a domain classifier that is executed in a typical adversarial way, where the classifier tries to determine which domain the domain-invariant features come from, while other modules try to deceive the classifier. Qu et al. (2019) implemented category-level alignment on the premise of global marginal alignment to generate more discriminative features. Li et al. (2019b) proposed selective adversarial learning (SAL) method to perform local semantic alignment of fine-grained domain adaptation, so as to obtain a better sharing feature space.

Most current works regarded the ABSA task as a domain-dependent problem. Existing domain adaption methods can only solve the problem of data scarcity in some specific domains, so it’s interesting to improve the model’s generalization ability and make it adapt to multiple domains simultaneously.

### Multimodal sentiment analysis

Multimodal sentiment analysis aims to identify sentiment from multiple modals (*e.g.*, text, image, video), its key challenge is how to fusion the data from different modals. There are mainly three combination strategies: early fusion, intermediate fusion and late fusion. Specifically, early fusion directly integrates multiple sources of data into a single feature vector, late-fusion aggregates the decisions from multiple sentiment classifiers which are trained by only single modal data, intermediate fusion’s fusion process is conducted in the intermediate layers of the neural networks (Huang et al., 2019).

Most studies adopt Intermediate fusion to integrate the information. Huang et al. (2019) integrated intermediate fusion and late fusion into a unified framework to exploit the discriminative features in texts and images, then explore the internal correlations between different modalities. Truong & Lauw (2019) proposed a visual aspect attention network (VistaNet), which uses images as the attention and uses visual information as the alignment of sentence level. Chen et al. (2017a) performed modality fusion at word-level and proposed gated multimodal embedding LSTM with temporal attention model (GME-LSTM(A)), which used gating mechanism trained using reinforcement learning. Xu, Mao & Chen (2019) proposed multi-interactive memory network (MIMN), where two interactive memory networks capture the multiple interactive influences between textual and visual information.

An idea may come up naturally that we can improve the model’s performance by integrating more modalities, but it may introduce noise when modalities convey the opposite sentiment. Building some practicable principles helps us identify which modal data is useful may be a good idea to handle these complex scenarios.

### Twitter sentiment analysis

Informal texts don’t have a unified structure and many omissions (*e.g.*, subject, verb, object) and ambiguities (*e.g.*, perhaps, perhaps) may appear, therefore, working with informal text on social media(*e.g.*, Twitter) is a new challenge. The SemEval series dataset also provides specialized datasets for the Twitter sentiment analysis task (*e.g.*, SemEval-2014 task 9 (Rosenthal et al., 2014), SemEval-2015 task 10 (Rosenthal et al., 2015), SemEval-2016 task 4 (Nakov et al., 2016) and SemEval-2017 task 4 (Rosenthal, Farra & Nakov, 2017)).

There are also some works on Twitter sentiment analysis. Vosoughi, Zhou & Roy (2015) analyzed the variation of tweet sentiments across time, authors, locations and used a Bayesian approach to incorporate the relationship between these factors and tweet sentiments into standard n-gram based Twitter sentiment classification. Caragea, Dinu & Dumitru (2018) explored a range of deep learning models at both Twitter and user level and studied the verb tense usage as well as the presence of polarity words in optimistic and pessimistic Twitter.

### Multilingual application

With the popularity of social platforms, the focus of sentiment analysis has shifted from English to multilingualism. Peng et al. (2018a) studied Chinese aspect from three different levels of granularity: radicals, characters and words. Dashtipour et al. (2020) proposed a concept-level hybrid framework with linguistic rules and deep learning methods for Persian sentiment analysis. Elshakankery & Ahmed (2019) proposed a hybrid incremental learning method (HILATSA) based on lexicon and machine learning for Arabic Twitter sentiment analysis. Pasupa & Seneewong Na Ayutthaya (2019) compared the performance of several traditional deep learning models on Thai children’s stories. In summary, the challenge of multilingualism is to find the features of specific language (*e.g.*, semantic and syntactic).

### Future expectations

According to the above discussion, we can find that the research direction of aspect-based sentiment analysis mainly includes two aspects: on the one hand, the research of basic methods; on the other hand, the improvement of task generalization ability under different task scenarios.

For the study of ABSA task basic methods, knowledge accumulation becomes very important with the continuous mining of rich semantic information in the text. The knowledge graph is conducive to language understanding and can be widely paid attention to in the future.

In order to improve the generalization ability of tasks in different task scenarios, the existing methods are studied from the perspectives of the platform (*e.g.*, e-commerce platform, social media platform), language (*e.g.*, Chinese, English), and expression (explicit and implicit). Future research is likely to focus on increasingly complex real-world scenarios.

## Summary

With the rapid development of the Internet, more and more people express their opinions on the Internet, which led to an explosion of textual data, it is more difficult for us to obtain realistic and valuable information from these data.

This article provides an overview of deep learning for aspect-based sentiment analysis and describes the ABSA task in detail from two different perspectives, *i.e.*, subtasks of ABSA and task modeling process. Unlike previous work, we re-division the ABSA task into IE task and SC task, where the IE task can be further classified into ATE task, OTE task and AOTCE task. According to the process of task modeling, the ABSA task also can be divided into pipeline ABSA and multi-task ABSA, where pipeline ABSA executed subtasks serially and multi-task ABSA executed subtasks parallelly.

In this article, we focused on the relations between different targets. Specifically, there are many complex relations between different targets, which contain rich information (*e.g.*, semantics information, sentimental information, syntax information, location information). In the pipeline ABSA, we divided the task into ATE task, OTE task, AOTCE task and SC task. For the ATE task, we explore the relations between aspects/aspect categories and context; for the OTE task, we explore the relations between opinions and context; for the AOTCE task, we explore the relations between ATE task and OTE task; for the SC task, we explore the relations among aspect, context and language structure. In the multi-task ABSA, we explore the relations between its subtasks and present some approaches to representing the ABSA task in a unified framework. The goal of the above tasks is to obtain high-level discriminative feature representations or enriched embedding representations.

To conclude, ABSA based on deep learning methods is still in the initial stage. With the deepening of the research, people will gradually begin to pay attention to the relations between different targets. We believe that in the future more research will constantly explore new targets and new relations. For example, the constituent tree and dependency tree have not been fully utilized. Moreover, when the information contained in the reviews has been fully explored, how to obtain new information from external knowledge (*e.g.*, knowledge graph) is also the direction of future research.

## List of Abbreviations

ABSA: Aspect-Based Sentiment Analysis
ACD: Aspect Category Detection
ACSA: Aspect Category Sentiment Analysis
AOPE: Aspect-Opinion Pair Extraction
AOTCE: Aspect-Opinion Term Co-Extraction
ASPE: Aspect Sentiment Pair Extraction
ASQE: Aspect Sentiment Quadruple Extraction
ASTE: Aspect Sentiment Triple Extraction
ATE: Aspect Term Extraction
BiGRU: Bidirectional Gated Recurrent Unit
BiRNN: Bidirectional Recurrent Neural Network
CNN: Convolutional Neural Networks
CV: Computer Vision
CRF: conditional random field
DL: Deep Learning
DNN: Deep Neural Networks
FN: False Negative
FP: False Positive
GAT: Graph Attention Network
GCN: Graph Convolutional Networks
GNN: Graph Neural Network
GRU: Gated Recurrent Unit
IE: Information Extraction
LSTM: Long Short-Term Memory
MemNN: Memory Network
MRC: Machine Reading Comprehension
NLI: natural language inference
NLP: Natural Language Processing
NN: Neural Network
OE: Opinion target Extraction
OOV: Out Of Vocabulary
OTE: Opinion Term Extraction
QA: Question Answering
RNN: Recurrent Neural Network
SA: Sentiment Analysis
SC: Sentiment Classification
SP: Sentiment Polarity
Seq2Seq: Sequence to Sequence
TN: True Negative
TOWE: Target-oriented Opinion Words Extraction
TP: True Positive

## Additional Information and Declarations

### Data Availability

The following information was supplied regarding data availability: This is a literature review.
